# Supplementary material for: Development of Methotrexate Complexes Endowed with New Biological Properties Envisioned for Musculoskeletal Regeneration in Rheumatoid Arthritis Environments
Source: Int J Mol Sci. 2022 Sep 2;23(17):10054. doi: 10.3390/ijms231710054 (PMC9456012; doi:10.3390/ijms231710054)
Supplement: Supplementary file 1 [file ijms-23-10054-s001.zip › ijms-1817741-supplementary.pdf]

Supplementary Materials (SM)

Development of methotrexate complexes endowed with new biological properties envisioned for musculoskeletal regeneration in rheumatoid arthritis environments

Daniel Fernández-Villa [1,2], Rosa Ana Ramírez [1,2], Inmaculada Aranaz [3], Niuris Acosta [3], Blanca Vázquez-Lasa [1, 2] and Luis Rojo [1, 2, *]

[1] Instituto de Ciencia y Tecnología de Polímeros (ICTP)CSIC, 28006 Madrid, Spain

[2] Centro de Investigación Biomédica en Red de Bioingeniería, Biomateriales y Nanomedicina (CIBER-BBN), 28029, Madrid, Spain

[3] Instituto Pluridisciplinar, Departamento de Química en Ciencias Farmacéuticas, Facultad de Farmacia, Universidad Complutense de Madrid, 28040, Madrid, Spain

* Correspondence: rojodelolmo@ictp.csic.es (L.R.); Tel.: +34-915-622-900

Supplementary Tables

Table S1. TGA main thermal stability results for MTX and its derivatives.

Drug	T_{max} 1st step (^{o}C)	T_{max} 2nd step (^{o}C)	$T_{50\%}$ (^{o}C)	Mass at 600 ^{o}C (%)
**MTX**	78	250	332	34.39
**SrMTX**	69	359	476	43.87
**ZnMTX**	143	319	> 650	56.14
**MgMTX**	45	332	432	38.42

Abbrev: $T_{50\%}$ = Temperature for a 50% weight loss

Table S2. ATR-FTIR main frequency (cm^{-1}) assignments of MTX and its derivatives.

Assignment	MTX	SrMTX	ZnMTX	MgMTX
**⚜(COOH)**	1682	N/A	N/A	N/A
**⚜$_{asy}$ (COO^-)**	1600; 1494	1601; 1500 (↑)	1603; 1508 (↑)	1603; 1511 (↑)
**⚜$_{sy}$ (COO^-)**	1450	1444 (↓)	1446 (↓)	1448 (↓)
**⚜⚜⚜⚜asy-sy⚜**	150	157(↑)	157 (↑)	155 (↑)
**⚜(CN)**	1206	1206	1206	1206

Abbrev: N/A = not applicable

Table S3. Non-linear regression analyses of cytotoxicity studies and ANOVA tests

Cytotoxicity	Cell Line	MTX	SrMTX	ZnMTX		MgMTX
				MTX	Zn^{2+}	
**EC50 (mM)**	RAW264.7	0.1748 ± 0.0351	0.1673 ± 0.0251	0.1253 ± 0.0629	219.7 ± 3.95	0.2492 ± 0.0698
	Sig.	*a*	*a*	*a*	*b*	*a*
**IC50 (mM)**	RAW264.7	0.7204 ± 0.3076	0.5569 ± 0.15765	0.4317 ± Ind.		N/A
	Sig.	*c*	*c*	*c*		N/A
	HC-a cells	N/A	N/A	66.62 ± 0.92		N/A
	Sig.	N/A	N/A	N/A		N/A

Abbrev: N/A = not applicable; Sig. = Significance; Ind. = Indeterminate. Mean values and standard deviations of EC50 and IC50 calculated by non-linear regression analyses are shown. Results from one-way ANOVA tests comparing among conditions are included. Different letters denote significant differences ($P < 0.05$, Tuckey post hoc test).

Supplementary Figures

(A)

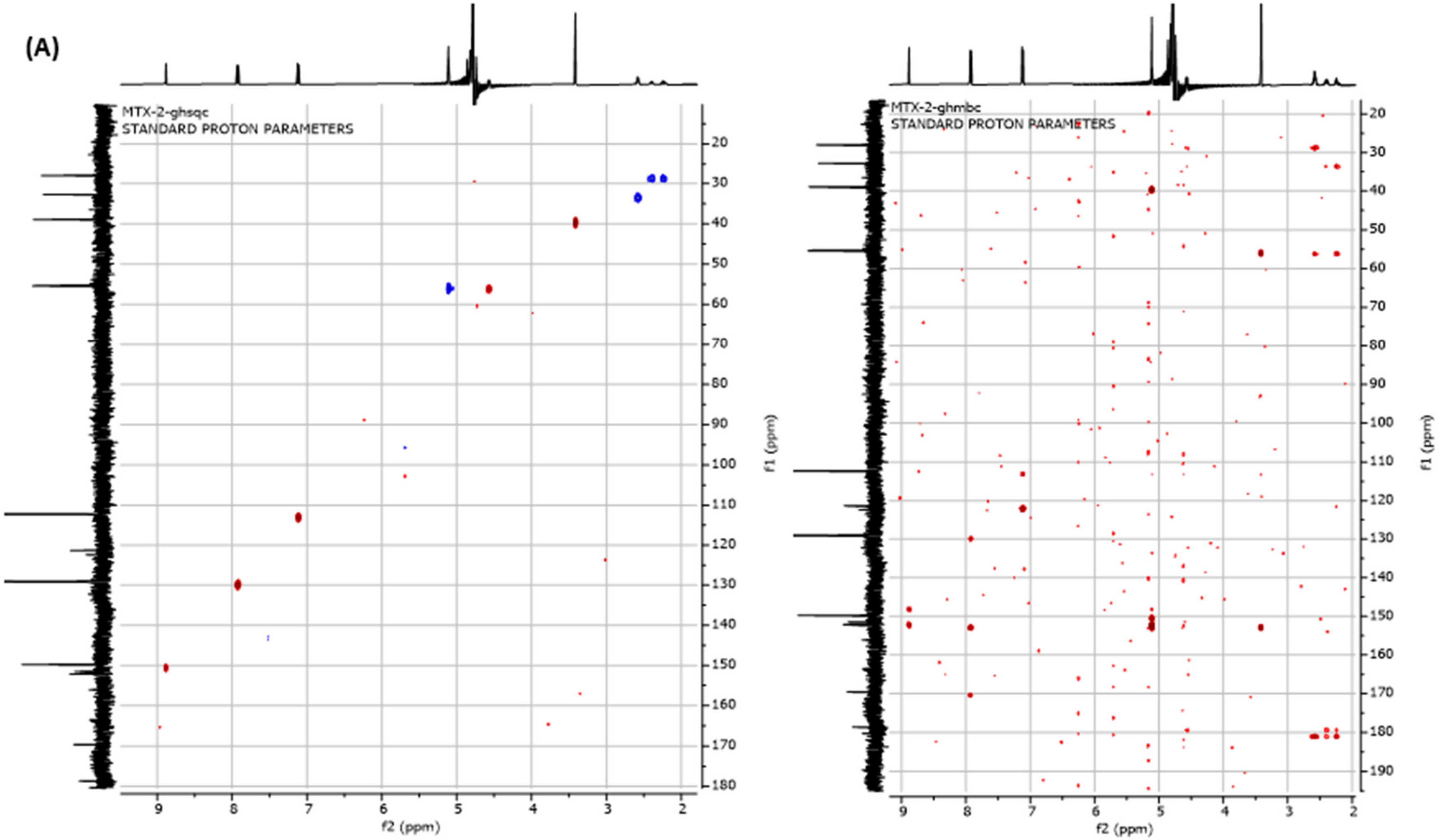

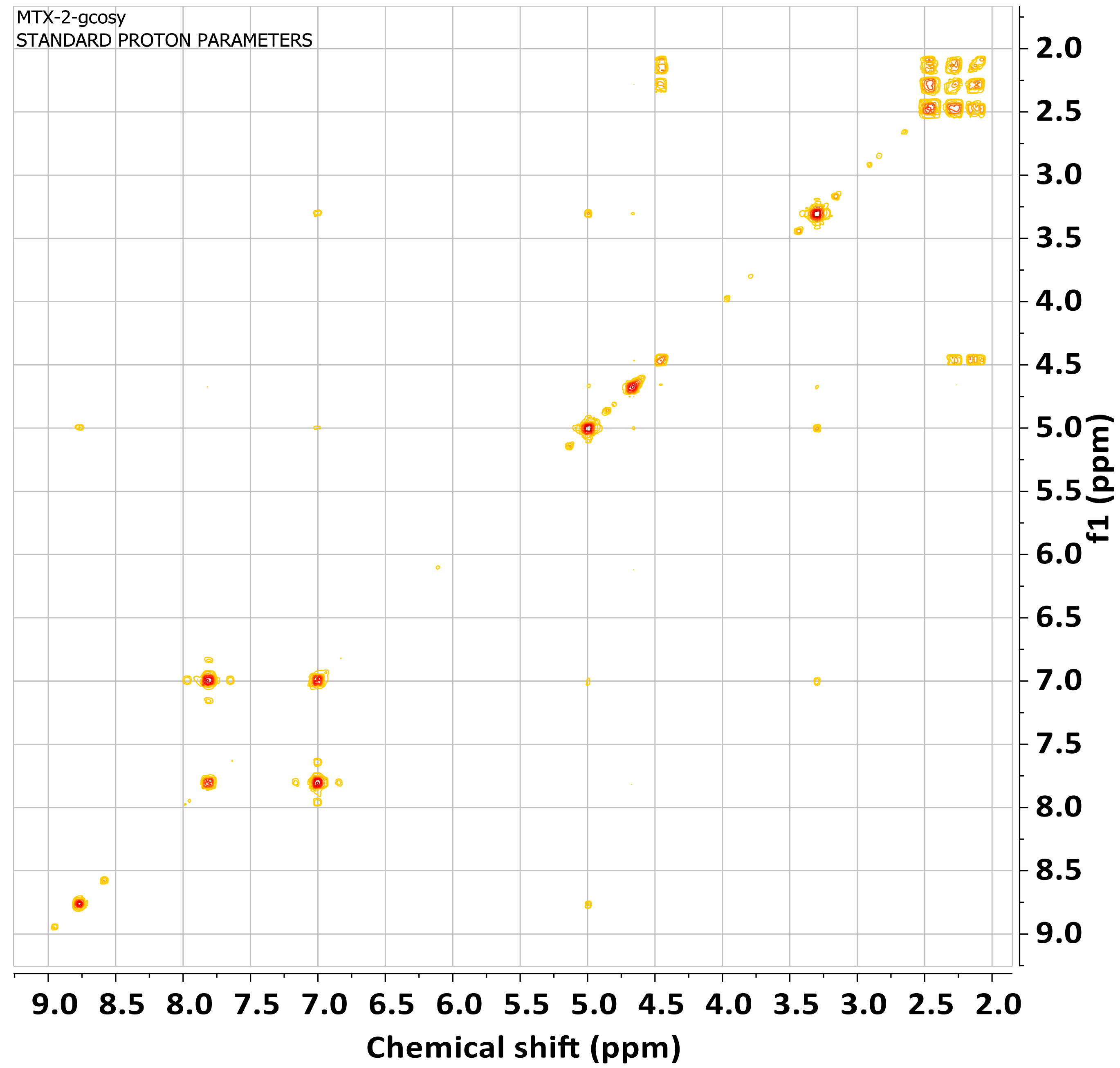
MTX-2-gcosy
STANDARD PROTON PARAMETERS
9.0 8.5 8.0 7.5 7.0 6.5 6.0 5.5 5.0 4.5 4.0 3.5 3.0 2.5 2.0
Chemical shift (ppm)
f1 (ppm)

(B)

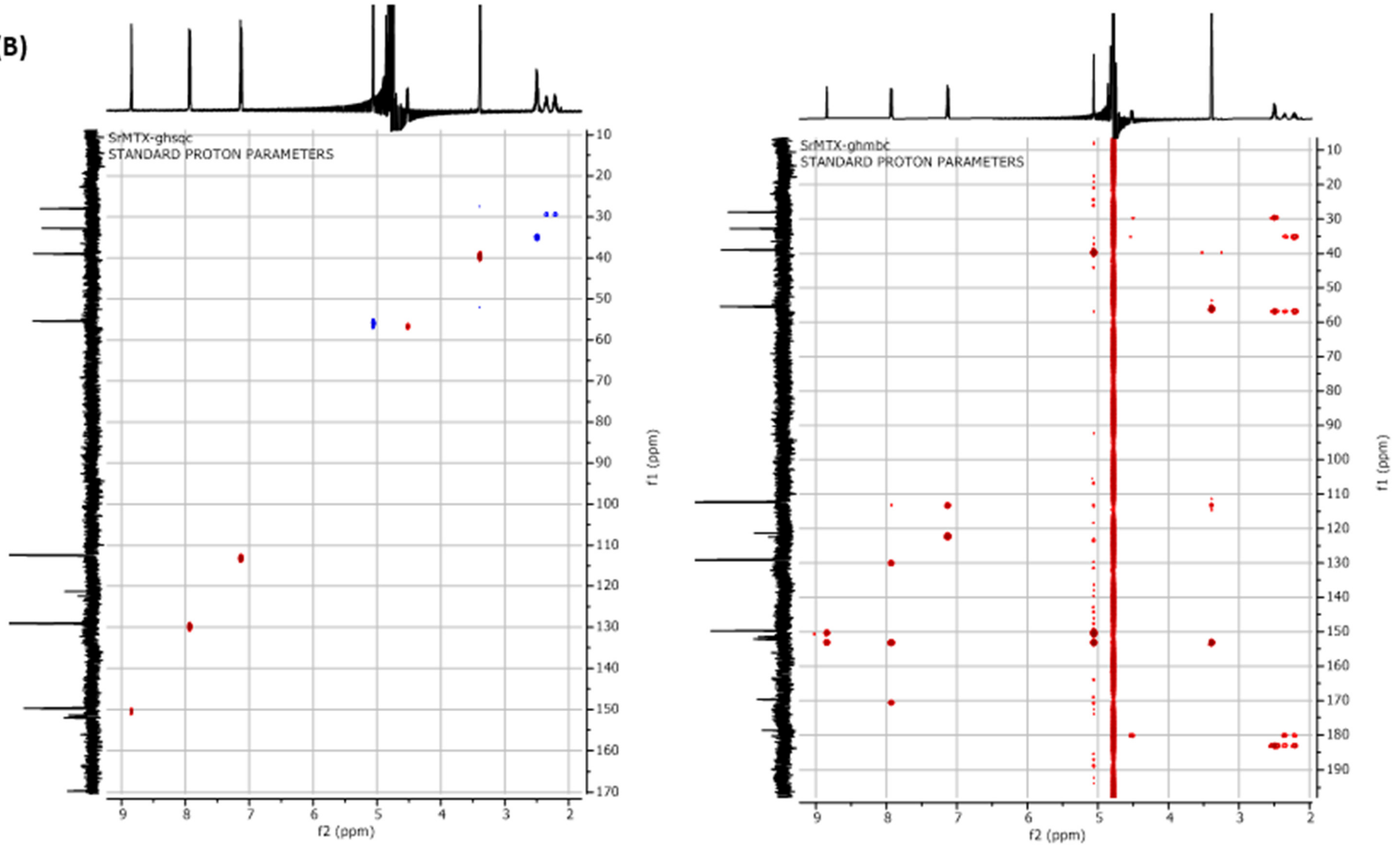
SrMTX-ghsqc
STANDARD PROTON PARAMETERS
f1 (ppm)
f2 (ppm)
SrMTX-ghmbc
STANDARD PROTON PARAMETERS
f1 (ppm)
f2 (ppm)

(C)

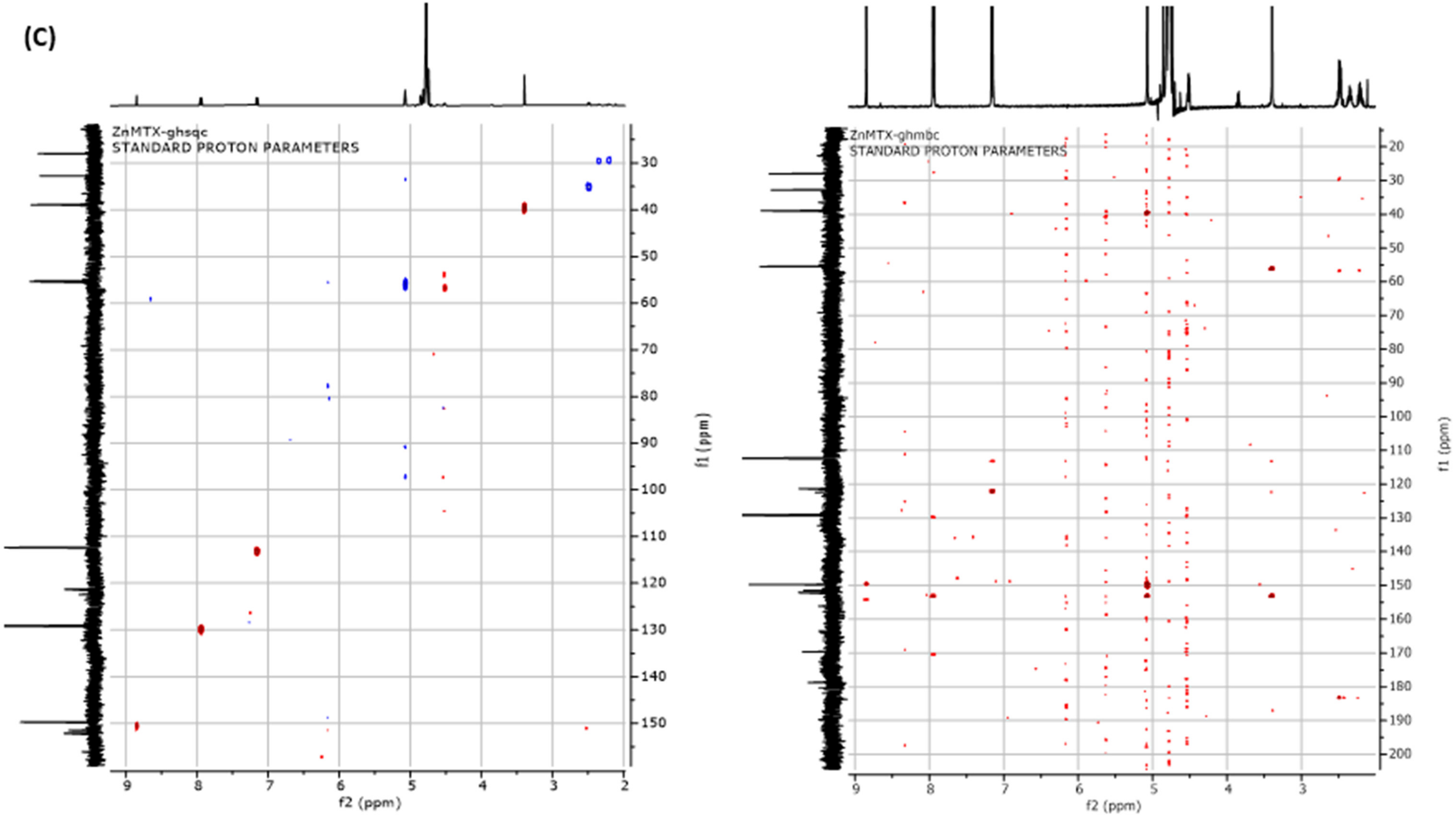

ZnMTX-ghsqc
STANDARD PROTON PARAMETERS
f2 (ppm)
f1 (ppm)
ZnMTX-ghmbc
STANDARD PROTON PARAMETERS
f2 (ppm)
f1 (ppm)

(D)

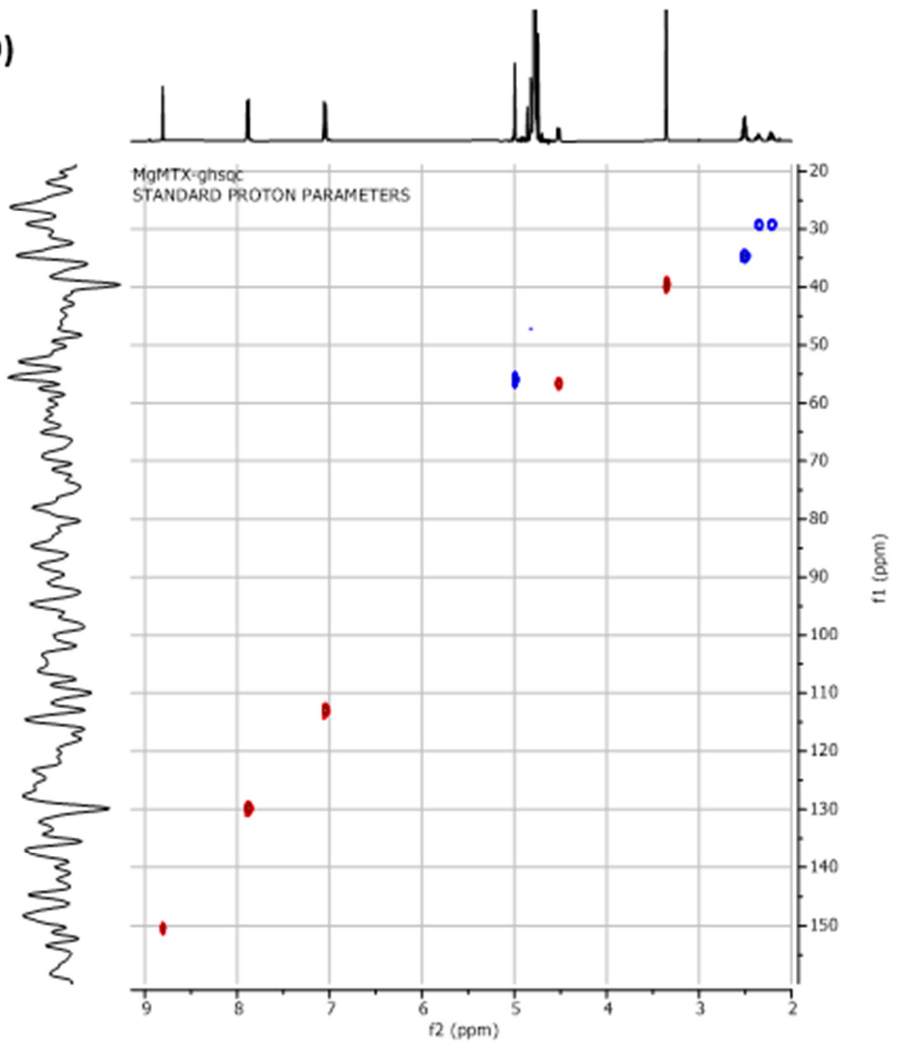
MgMTX-ghsqc
STANDARD PROTON PARAMETERS
f2 (ppm)
f1 (ppm)

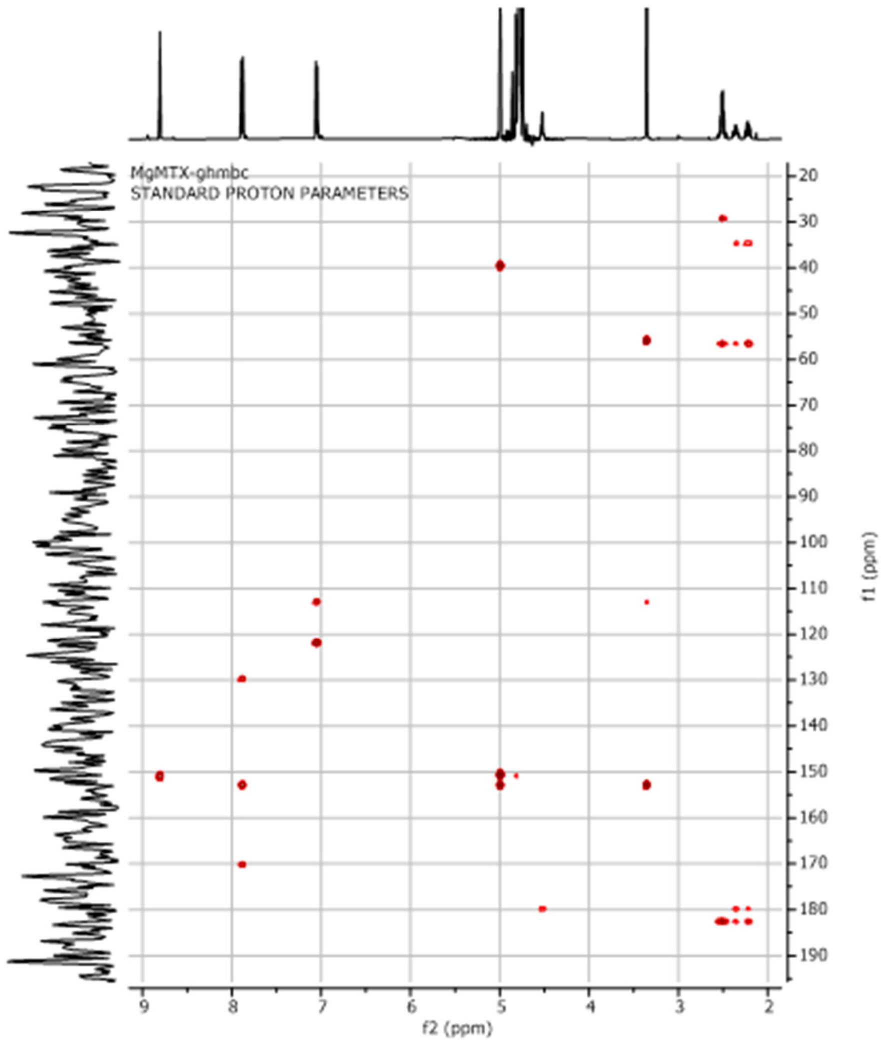
MgMTX-ghmbc
STANDARD PROTON PARAMETERS
f2 (ppm)
f1 (ppm)

(E)

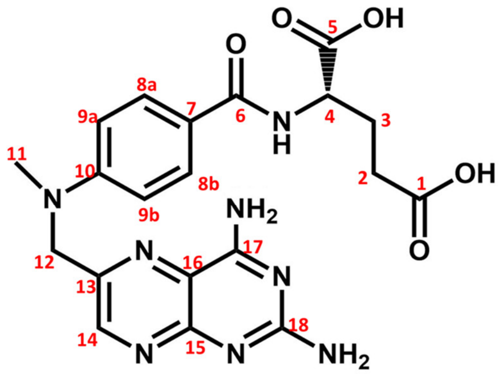

Colour Legend ^{13}C
|X| > 1
0,5 < |X| < 1
0,2 < |X| < 0,5
|X| < 0,2

	MTX		SrMTX		ZnMTX		MgMTX	
Position	δ(^{1}H) (ppm)	δ(^{13}C) (ppm)	δ(^{1}H) (ppm)	δ(^{13}C) (ppm)	δ(^{1}H) (ppm)	δ(^{13}C) (ppm)	δ(^{1}H) (ppm)	δ(^{13}C) (ppm)
1	N/A	181.005	N/A	183.022	N/A	183.03	N/A	182.598
2	2.578	33.496	2.494	34.863	2.489	35.058	2.504	34.668
3	2.239 2.382	28.711	2.209 2.344	29.395	2.207 2.343	29.394	2.213 2.353	29.199
4	4.567	56.15	4.518	56.736	4.513	56.736	4.518	56.541
5	N/A	179.364	N/A	179.975	N/A	n.i.	N/A	179.785
6	N/A	170.457	N/A	170.599	N/A	170.372	N/A	170.174
7	N/A	122.17	N/A	122.312	N/A	122.085	N/A	121.887
8	7.925	129.778	7.929	129.778	7.945	129.778	7.878	129.778
9	7.113	113.178	7.13	113.178	7.155	113.178	7.047	112.983
10	N/A	152.877	N/A	153.253	N/A	153.027	N/A	152.829
11	3.412	39.745	3.389	39.55	3.395	39.55	3.351	39.55
12	5.111	55.955	5.06	55.955	5.073	56.151	4.992	55.955
13	N/A	152.174	N/A	153.019	N/A	154.199	N/A	151.261
14	8.881	150.48	8.845	150.48	8.849	150.48	8.803	150.48
15	N/A	148.189	N/A	150.206	N/A	149.51	N/A	150.374
16	n.i.	n.i.	n.i.	n.i.	n.i.	n.i.	n.i.	n.i.
17	n.i.	n.i.	n.i.	n.i.	n.i.	n.i.	n.i.	n.i.
18	n.i.	n.i.	n.i.	n.i.	n.i.	n.i.	n.i.	n.i.

	SrMTX	ZnMTX	MgMTX
Position	Δδ(^{13}C) (ppm)	Δδ(^{13}C) (ppm)	Δδ(^{13}C) (ppm)
1	2.017	2.025	1.593
2	1.367	1.562	1.172
3	0.684	0.683	0.488
4	0.586	0.586	0.391
5	0.611	n.i	0.421
6	0.142	0.085	0.283
7	0.142	0.085	0.283
8	0	0	0
9	0	0	0.195
10	0.376	0.15	0.048
11	0.195	0.195	0.195
12	0	0.196	0
13	0.845	2.025	0.913
14	0	0	0
15	2.017	1.321	2.185
16	n.i.	n.i.	n.i.
17	n.i.	n.i.	n.i.
18	n.i.	n.i.	n.i.

Figure S1. 2D-NMR spectroscopy analyses (gHSQC, gHMBC and gCOSY), ^{1}H and ^{13}C assignations and their corresponding chemical shifts. A) gHSQC, gHMBC and gCOSY of MTX; B) gHSQC and gHMBC of SrMTX; C) gHSQC and gHMBC of ZnMTX; D) gHSQC and gHMBC of MgMTX; E) MTX molecular structure, ^{1}H and ^{13}C assignations with their corresponding chemical shifts, and their changes between MTX and its derivatives. Abbrev: n.i. = not identified.
